# Variation of the factor H-binding protein of *Neisseria meningitidis*

**DOI:** 10.1099/mic.0.027995-0

**Published:** 2009-12

**Authors:** Carina Brehony, Daniel J. Wilson, Martin C. J. Maiden

**Affiliations:** 1 Department of Zoology, University of Oxford, OX1 3PS, UK; 2 Department of Human Genetics, University of Chicago, 920 East 58th Street, CLSC #410, Chicago, IL 60637, USA

## Abstract

There is currently no comprehensive meningococcal vaccine, due to difficulties in immunizing against organisms expressing serogroup B capsules. To address this problem, subcapsular antigens, particularly the outer-membrane proteins (OMPs), are being investigated as candidate vaccine components. If immunogenic, however, such antigens are often antigenically variable, and knowledge of the extent and structuring of this diversity is an essential part of vaccine formulation. Factor H-binding protein (fHbp) is one such protein and is included in two vaccines under development. A survey of the diversity of the fHbp gene and the encoded protein in a representative sample of meningococcal isolates confirmed that variability in this protein is structured into two or three major groups, each with a substantial number of alleles that have some association with meningococcal clonal complexes and serogroups. A unified nomenclature scheme was devised to catalogue this diversity. Analysis of recombination and selection on the allele sequences demonstrated that parts of the gene are subject to positive selection, consistent with immune selection on the protein generating antigenic variation, particularly in the C-terminal region of the peptide sequence. The highest levels of selection were observed in regions corresponding to epitopes recognized by previously described bactericidal monoclonal antibodies.

## INTRODUCTION

Meningococcal disease, caused by the Gram-negative bacterium *Neisseria meningitidis*, is an important cause of morbidity and mortality worldwide, with most disease being due to meningococci expressing one of five capsular polysaccharide antigens corresponding to serogroups A, B, C, Y and W135. Although serogroup B meningococci are a major cause of disease, particularly in industrialized countries (Jones, 1995; Pollard *et al.*, 2001; Trotter *et al.*, 2007), there is currently no vaccine against them due to the poor immunogenicity of the serogroup B capsular polysaccharide. This may be a consequence of its similarity to host antigens, which also raises concerns as to the safety of serogroup B polysaccharide as a vaccine component (Finne *et al.*, 1983). A variety of subcapsular cellular components, particularly outer-membrane proteins (OMPs), have been evaluated as possible alternative vaccine antigens (Jodar *et al.*, 2002). These have included outer-membrane vesicle (OMV) vaccines that contain PorA, which have been used to target single-clone epidemics of meningococcal disease in Cuba, Norway and New Zealand with some success (Bjune *et al.*, 1991; O'Hallahan *et al.*, 2005; Rodriguez *et al.*, 1999). A major issue is the antigenic variability of OMPs, which complicates the development of vaccines with broad coverage. PorA, for example, has two major regions of antigenic variability (Russell *et al.*, 2004) with 188 and 517 different peptide sequences in each region described by January 2009 (http://neisseria.org/nm/typing/).

Wider coverage against variable antigens can be attained by inclusion of multiple variants in vaccine formulations, and multivalent PorA vaccines such as NonaMen have been developed (van den Dobbelsteen *et al.*, 2007). For vaccine formulations such as this, it is necessary to have detailed molecular epidemiological information about the current and most frequently occurring strains in the population. Alternatively, more conserved antigens, such as *Neisseria* adhesin A (NadA) and *Neisseria* surface protein A (NspA) (Comanducci *et al.*, 2002; Martin *et al.*, 1997) and, despite its variability (Rokbi *et al.*, 2000), transferrin-binding protein B (TbpB) (Ala'Aldeen & Borriello, 1996), have been considered as candidates.

The vaccine candidate lipoprotein 2086 (LP2086) was discovered by an iterative process of immunization following differential detergent extraction and protein purification (Bernfield *et al.*, 2002; Fletcher *et al.*, 2004). It was also identified as genome-derived neisserial antigen 1870 (GNA1870) (Masignani *et al.*, 2003) by the technique known as ‘reverse vaccinology’ (Rappuoli, 2000). Subsequently, it has been given the name factor H-binding protein (fHbp) because of its role in modulating the activity of the alternative complement pathway, where it binds the regulatory protein factor H (fH) (Madico *et al.*, 2006). fH has a critical role in maintaining homeostasis of the complement system and also, by attachment to host cells and tissue, in preventing potential damage to them by inhibiting complement activation (Rodríguez de Córdoba *et al.*, 2004). Several organisms, including *N. meningitidis*, mimic human tissue by recruiting fH and coating their surface, thereby avoiding complement-mediated lysis (Lambris *et al.*, 2008; Schneider *et al.*, 2006). In the case of the meningococcus, fHbp is the only receptor for fH on its surface (Schneider *et al.*, 2009). The protein is present in all meningococci, although levels of expression may vary in different isolates (Fletcher *et al.*, 2004; Masignani *et al.*, 2003). In comparison with other vaccine antigens, it is relatively sparse in its epitope surface-exposure in most meningococcal strains (Welsch *et al.*, 2004). Expression of fHbp has been found to be key for survival in *ex vivo* human blood and human serum, particularly in high-expressing strains (Seib *et al.*, 2009; Welsch *et al.*, 2008). Structurally, fHbp is a surface-exposed 29 kDa globular lipoprotein composed of two *β*-barrels connected by a short linker and is bound to the outer membrane by an N-terminal lipid anchor (Cantini *et al.*, 2009; Mascioni *et al.*, 2009; Schneider *et al.*, 2009). Recent analysis of the fH–fHbp interaction indicates that the fH recognition site spans the whole surface of fHbp and that previously described bactericidal epitope sites do not lie in this region, but epitopes that bind to antibodies that affect fH binding are found around the edge of the site (Schneider *et al.*, 2009).

The protein is a principal component of two recombinant protein vaccines in clinical trials at the time of writing. It is unique as a vaccine candidate in that it is able to elicit serum antibodies that activate classical complement pathway bacteriolysis and also prevent fH binding to the meningococcal cell surface, thus making it more susceptible to bactericidal activity (Madico *et al.*, 2006; Welsch *et al.*, 2008). Like the related human-restricted organism *Neisseria gonorrhoeae*, there is specificity of binding to human fH (Granoff *et al.*, 2009; Ngampasutadol *et al.*, 2008). This may help to explain the higher bactericidal titres obtained when using vaccine-induced antibodies with rabbit complement versus human complement and also the organisms' exclusively human-related pathogenicity.

In the present study, variation of fHbp in a reference set of diverse meningococcal isolates was surveyed. The association of particular variants with clonal complex and serogroup was established, and the levels of recombination and selection acting on it were determined. Also, a novel, unified nomenclature scheme was developed that was independent of subfamily/variant and a Web-accessible database established to facilitate querying of sequences and submission of new allele sequences.

## METHODS

### Isolates.

The 107 meningococci surveyed were representative of bacteria isolated worldwide in the latter half of the twentieth century, obtained from both patients with meningococcal disease and carriers (Maiden *et al.*, 1998). Two previously published *N. gonorrhoeae* protein sequences: YP_002793564 and EEH61327, from GenBank were also used as part of the analysis.

### Amplification of the fHbp gene and nucleotide sequence determination.

Amplification of an approximately 900 bp region including the fHbp gene and immediately flanking regions was carried out using the Long 5UNI 2086 and 3UNI pair of primers (Fletcher *et al.*, 2004). The PCRs were performed in 50 μl amplification reaction volumes using *Taq* polymerase (Qiagen) with 33 cycles of 95 °C for 50 s, 59 °C for 50 s, and 72 °C for 50 s with a final extension step of 72 °C for 7 min. The amplicons were purified by 20 % PEG/2.5 M NaCl precipitation and then used as templates for 10 μl dideoxynucleotide sequencing reactions using BigDye Ready Reaction Mix (Applied Biosystems). Oligonucleotide primers specific for each of the subfamilies A and B were used to amplify internal fragments of the purified amplified gene products: (for subfamily A) 5′2086forseq (5′-TAT GAC TAG GAG CAA ACC TG-3′), 3′2086forseq (5′-TAC TGT TTG CCG GCG ATG-3′), 2086interforseq5′LA primer (5′-AGC TCA TTA CCT TGG AGA GCG GA-3′); (for subfamily B) 2086seq3′BLA primer (5′-TTC GGA CGG CAT TTT CAC AAT GG-3′) and 2086seqBinternal (5′-GGC GAT TTC AAA TGT TCG ATT T-3′). Cycling conditions were 30 cycles of 96 °C for 10 s, 50 °C for 5 s and 60 °C for 4 min. Separation of the labelled extension products was carried out on a 3730 capillary DNA analyser (Applied Biosystems) at the Department of Zoology Sequencing Facility, University of Oxford.

### Analysis of sequence data.

Assembly and editing of nucleotide sequence data were carried out using the Staden suite of software (Staden, 1996). Reformatted nucleotide sequences were visualized, aligned and translated manually using SeqLab, part of the GCG Wisconsin Package (Womble, 2000) [Version 10.3 for Unix (Accelrys)]. The alignment was based on amino acid sequence similarity with codon integrity maintained. A web-based front end to the NRDB program (written by Warren Gish, Washington University) was used to compare nucleotide and amino acid sequences to find those that were identical (http://pubmlst.org/analysis/).

The mega 3.1 program (Kumar *et al.*, 2004) was used to calculate the overall mean distances as well as the within- and between-group distances for sequences using the Kimura two-parameter model for nucleotide sequences and *p*-distances for amino acid sequences, and also to produce distance matrix-based neighbour-joining trees. The reliability of the inferred trees was assessed by the bootstrap test with 2000 replications. mega 3.1 implements Felsenstein's bootstrap test evaluated using Efron's bootstrap resampling method. In the bootstrap test of phylogeny, a matrix of *m* sequences×*n* (nucleotides/peptides) is sampled with replacement (bootstrapping). These new sequences are reconstructed into a tree using the previously used phylogenetic method and the topology is compared with the original tree. This procedure is repeated 2000 times, and the percentage of times a particular interior branch is the same between the original tree and the bootstrap tree is given. Boostrapping is a means of assessing confidence in a particular phylogeny, and values are interpreted as the probability of interior branches being ‘correct’ (generally 95 % or higher).

The software package clonalframe version 1.1, which implements a statistical model for inferring bacterial microevolution, was used for phylogenetic analysis and to identify regions likely to have undergone homologous recombination (Didelot & Falush, 2007). clonalframe performs inference in a Bayesian framework which assumes a standard neutral coalescent model whereby the bacteria in the sample come from a constant-sized population in which each bacterium is equally likely to reproduce, irrespective of its previous history. The key assumption is that recombination events introduce a constant rate of substitutions to a contiguous region of sequence. Six independent runs, each with 250 000 iterations, 100 000 burn-in iterations and with every hundredth tree sampled, were used to derive a 75 % majority-rule consensus tree. paup* version 4.0b10 for Unix (Swofford, 1998) was used to construct phylogenetic trees using the maximum-likelihood method. clonalframe and maximum-likelihood tree outputs were imported and further annotated in mega 3.1.

Associations between subfamily/allele and clonal complex/serogroup were analysed using Fisher's exact test with Bonferroni correction applied as appropriate, with calculations performed with the R program version 2.7.1 (http://www.r-project.org/). Simpson's index of diversity (*D*) was used to assess the level of diversity of each subfamily/variant. It gives the probability that any two randomly selected individuals drawn from an infinitely large community belong to different species, or in the case of this study, the probability that isolates drawn from a population belong to different allele types (Hunter & Gaston, 1988; Simpson, 1949). The bias-corrected form of the formula used is as follows:
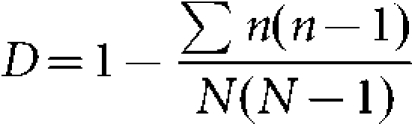
Where *N* is total number of isolates and *n* is total number of isolates of a particular genetic type. The value of the index ranges from zero to one, such that the nearer to one the greater the diversity and the nearer to zero the less the diversity. The 95 % confidence intervals (CIs) for these indices were calculated as described by Grundmann *et al.* (2001). Non-overlapping CIs indicate a significant difference in *D*.

### Analysis of selection pressures.

The start2 program (Jolley *et al.*, 2001) (http://pubmlst.org/software/analysis/start2/) was used for tests of recombination by the maximum chi-squared test and for selection using the ratio of non-synonymous to synonymous nucleotide substitutions (*d*_N_* *:* d*_S_ ratio). The omegamap program, which employs a Bayesian method to estimate the selection parameter *ω* (*d_N_ *:* d_S_*) and the recombination rate *ρ* from gene sequences by use of reversible jump Monte Carlo Markov Chain (Wilson & McVean, 2006), was used to detect selection and recombination by inferring the posterior distributions of *ω* and *ρ* along the gene. The means of the posterior distributions were used as a point estimates for *ω* and *ρ*. The per-site posterior probability of positive selection was also used to summarize the posterior distribution of *ω*. Three independent omegamap runs, each with 1 000 000 iterations and a thinning interval of 100, were compared to assess convergence and combined. Output from the omegamap runs was used to visualize possible selection acting on the sequence by means of fireplots and graphs indicating the posterior probability of positive selection along the sequence. Fireplots visualize the posterior probability on log(*ω*) or *ω* along the sequence using a colour gradient, where a higher posterior density is represented by more intense colour (closer to white) and lower posterior density is represented by less intense colour (closer to red). These plots were produced using the R program version 2.7.1 (http://www.r-project.org/). The point estimate of *ω* was used to colour a 3D pdb file of the solution structure of a complex between a subfamily B/variant 1 GNA1870/fHbp protein and a region of the fH protein (Schneider *et al.*, 2009) (http://www.rcsb.org/pdb/home/home.do Protein Data Bank code 2W81).

### fHbp database.

Unique nucleotide and peptide sequences were arbitrarily assigned allele numbers in order of discovery. A database was established containing these allele sequences obtained as part of this study, from direct submissions from collaborators or by interrogation of the GenBank database. agdbnet antigen sequence software for web-based bacterial typing was used to do this (http://pubmlst.org/software/database/agdbnet/). It allows simultaneous blast querying of multiple loci using either nucleotide or peptide sequences (Jolley & Maiden, 2006).

## RESULTS

### Diversity of fHbp gene and protein

The fHbp gene was found in all 107 isolates, and among these, a total of 28 unique gene sequences encoding 27 different amino acid sequences were identified (Table 1). Unique peptide and nucleotide sequences were arbitrarily assigned allele numbers in order of discovery and entered into a database (http://neisseria.org/nm/typing/fhbp/), providing a comprehensive repository of reported fHbp diversity.

**Fig. 1.**
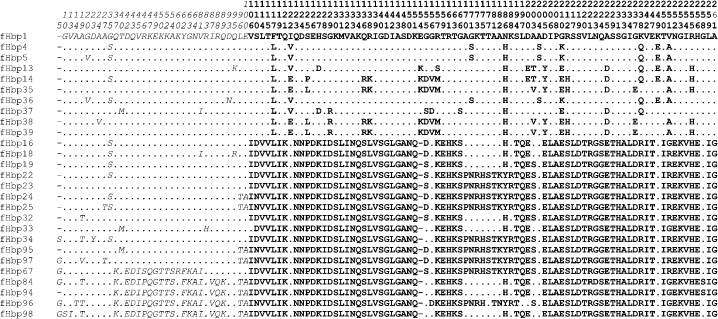
Aligned fHbp peptide allele variable sites. Alleles fHbp1, 4, 5, 13, 14 and 35–39 are subfamily B/variant 1; alleles fHbp16, 18, 19, 22–25, 32–34, 95 and 97 are subfamily A/variant 2; and alleles fHbp67, 84, 94, 96 and 98 are subfamily A/variant 3. The sequences in italic type indicate the N-terminal first 100 aa.

On the basis of 798 unambiguously aligned nucleotides there were a total of 299 variable nucleotide sites within these sequences (Fig. 1). Two broad groups of sequence variant types were evident from this alignment. This corresponds to the previously identified groups: subfamily A/variant 2 and subfamily B/variant 1 (Fletcher *et al.*, 2004). Within subfamily A/variant 2 there were five putative subfamily A/variant 3 sequences which differed from the rest of subfamily A/variant 2 mainly in the N-terminal first 100 amino acids (Masignani *et al.*, 2003). The subfamily A/variant 2 (including variant 3) was significantly more diverse in terms of allele types than subfamily B/variant 1 [*D*=0.91 (95 % CIs 0.87–0.95) versus 0.80 (95 % CIs 0.75–0.85), respectively].

There was 63 % nucleotide sequence identity shared between the two main groups and larger identity within them: 85 % nucleotide site identity within subfamily A/variant 2 and 87 % nucleotide site identity within subfamily B/variant 1. The overall mean Kimura two-parameter *p*-distance among all gene sequences was 0.165. The within-group mean *p*-distances were 0.046 for subfamily A/variant 2 and 0.032 for subfamily B/variant 1, with a mean *p*-distance between the two subfamilies/variants of 0.302. There was 56 % deduced amino acid sequence identity shared between the two groups. Within subfamily A/variant 2 there was 81 % amino acid site identity and within subfamily B/variant 1 there was 87 % amino acid site identity. The overall mean *p*-distance among the amino acid sequences was 0.17. The within-group mean *p*-distances for subfamily A/variant 2 and subfamily B/variant 1 were 0.052 and 0.038, respectively. The mean *p*-distance between the two subfamilies/variants was 0.31. Without the subfamily A/variant 3 sequences, subfamily A/variant 2 nucleotide sequence identity was 90 % and amino acid site identity was 88 %.

Sequence variability was found throughout the gene and encoded protein (Fig. 1). There was a marked difference in variability, however, between the N-terminal first ∼105 aa and the C-terminal region of ∼161 aa, where sequences were more variable. The amino acid sequence identity of the C-terminal region between the two groups was 48 %; however, there was more identity within the groups (subfamily A/variant 2, 87 %; subfamily B/variant 1, 84 %). For the N-terminal region there was 67 % amino acid identity between the two subfamilies/variants, and 70 % within subfamily A/variant 2 and 93 % within subfamily B/variant 1. In the absence of the subfamily A/variant 3 sequences, for subfamily A/variant 2, there was 90 % amino acid identity in the C-terminal region and 86 % in the N-terminal region.

The amino acid Glu/Lys at position 154 was present in subfamily B/variant 1 isolates but not subfamily A/variant 2 isolates (Fig. 1). There was an absence, in subfamily A/variant 2 isolates, of Arg^204^ (here at amino acid 212), considered to be key in antibody binding in the subfamily B/variant 1 antigen (Giuliani *et al.*, 2005), where it is substituted with serine. There were 81 % of subfamily B/variant 1 isolates that contained Arg^204^; the rest had a histidine residue at this position. Two subfamily B/variant 1 isolates (IDs EG328 and 528; peptide 37) had a substitution of a G for a T in the final stop codon and were thus extended for a further nine bases. The N-terminal region separates the subfamily A/variant 3 isolate sequences from the other subfamilies/variants (Fig. 1). Subfamily A/variant 3 sequences contained an insertion at amino acids 67–69 of lysine, aspartic acid and asparagine, not present in the other variants. This insertion has previously been noted as being present in a subset of subfamily A protein sequences (Fletcher *et al.*, 2004). Three of the five subfamily A/variant 3 sequences also contained a 5 aa glycine-rich insertion at the N-terminal end. This insertion has been found in sequences of both subfamilies/variants (Fletcher *et al.*, 2004), and is thought to be used as a means of lengthening the chain that attaches the folded protein to accommodate differences in lipooligosaccharide length on the outer membrane (Mascioni *et al.*, 2009).

**Fig. 2.**
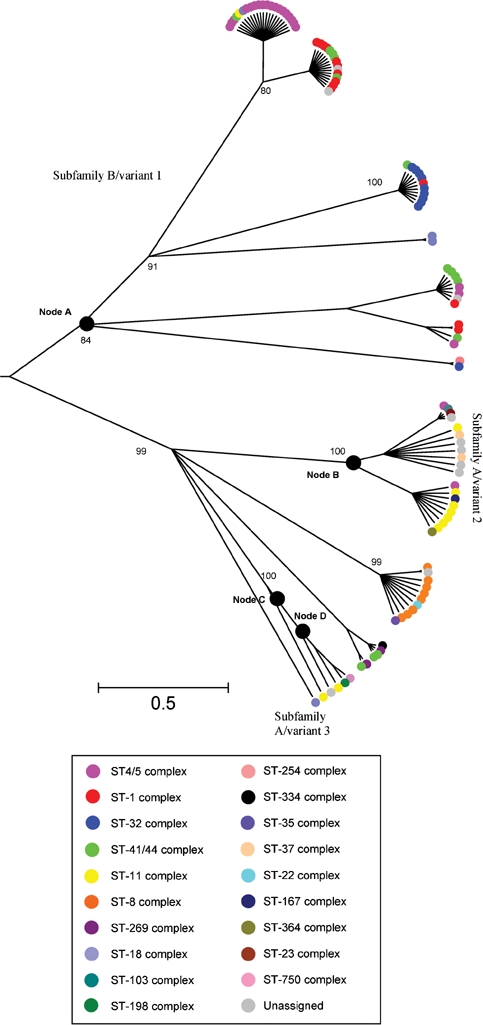
A 75 % majority-rule consensus clonalframe radial tree of 107 aligned nucleotide sequences with colour coding according to clonal complex and confidence values for nodes. A node is defined as the most recent common ancestor of the isolates in the branch above it.

Genealogical analysis using clonalframe, and phylogenies constructed with neighbour-joining (Supplementary Fig. S2) and maximum-likelihood methods (not shown), resolved protein and nucleotide sequences into two major groups, with the variant 3 isolates branching off from the rest of subfamily A/variant 2 (Fig. 2). clonalframe gives equal weight to genetic events that result in one nucleotide change, and single horizontal genetic exchange events that result in many nucleotide changes, and did not separate the putative subfamily A/variant 3 isolate sequences from the other subfamily A/variant 2 isolates (Fig. 2), although they were more distant from them in neighbour-joining (Supplementary Fig. S2) and maximum-likelihood phylogenies (data not shown).

### Clonal complex/serogroup association

The distribution of subfamily/variant alleles was not random among clonal complexes, showing some clustering with particular meningococcal genotypes (Fig. 2 and Supplementary Fig. S1). For example, the sequence type (ST)-11 complex was associated with subfamily A/variant 2 and in particular a cluster within this subfamily/variant (six of eight isolates were fHbp peptide 22; Fisher's exact test both *P*<0.005). Similarly, the ST-8 complex was found to be associated only with this subfamily/variant and seven of eight isolates had the fHbp 16 peptide (Fisher's exact test *P*<0.005). The serogroup A-associated complexes ST-4 and ST-5 were clustered together and associated mainly with subfamily B/variant 1. The ST-4 complex was particularly homogeneous as all isolates had the fHbp 5 peptide (Fisher's exact test *P*<0.005). All the ST-32 complex isolates were found associated with subfamily B/variant 1, with nine of 10 isolates having the fHbp 1 peptide type (Fisher's exact test *P*<0.005). The ST-1 complex was significantly associated with fHbp peptide 4 (10 of 14 isolates, Fisher's exact test *P*< 0.005). The other main hyperinvasive lineage, the ST-41/44 complex, was more diverse with respect to the subfamilies/variants observed.

Similarly, there was a relationship between serogroup (particularly non-B serogroups) and variants/subfamilies (Table 1), although this was at least in part due to the known association of clonal complex with serogroup (Trotter *et al.*, 2007). A total of 55 % of subfamily B/variant 1 were serogroup A (Fisher's exact test *P*<0.005) compared with 4 % for subfamily A/variant 2. Serogroup C was found in 1.6 % of subfamily B/variant 1, while it accounted for 35 % of subfamily A/variant 2 (Fisher's exact test *P*<0.005). There were no W-135, Y or Z subfamily B/variant 1 types, while each serogroup accounted for 4 % of subfamily A/variant 2 isolates. Serogroup B was more evenly distributed, accounting for 50 % of subfamily A/variant 2 isolates and 44 % of subfamily B/variant 1 isolates. However, 70 % of serogroup B disease-associated isolates were subfamily B/variant 1.

### Evidence of recombination and selection

**Fig. 3.**
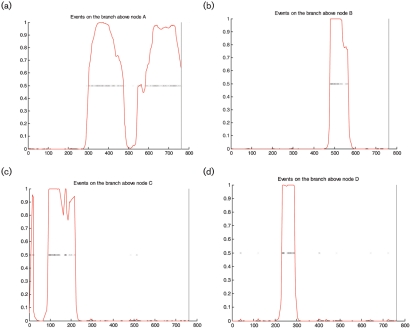
Representation of fHbp gene recombination events (a–d). The nucleotide sequence of the fHbp gene is on the *x* axis, with the red line indicating the probability for an import from 0 to 1 (*y* axis). The panels depict genetic events above nodes A, B, C and D shown in the 75 % majority-rule consensus clonalframe tree panel (Fig. 2). Each inferred substitution is indicated by a cross, the intensity of which indicates the posterior probability for that substitution. In (a), horizontal genetic exchange is depicted occurring from base 300 to base 500 and from base 550 to base 800; in (b), from base 450 to base 600; in (c), horizontal genetic exchange is depicted occurring from base 100 to base 250 and in (d) at about 200 and 300 bases.

Maximum chi squared analysis identified putative recombination sites after nucleotide sites 281 and 326. clonalframe analysis indicated strong evidence of horizontal genetic exchange in the C-terminal region from around 300 bp onwards (node A in Figs 2 and 3a). Also, in the N-terminal region of subfamily A/variant 3 sequences there was strong evidence of lateral gene transfer which presumably gave rise to this variant within the subfamily A/variant 2 group (nodes C and D, Fig. 3c, d). Other points of recombination were identified, including above node B, which contains subfamily A/variant 2 sequences (Fig. 3b).

**Fig. 4.**
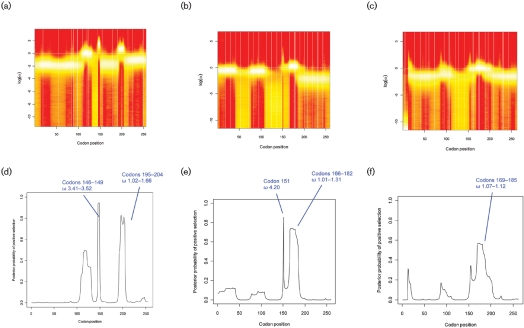
omegamap program output using subfamily A/variant 2 and subfamily B/variant 1 nucleotide sequences. (a), (b) and (c) depict fireplots of the sitewise posterior distribution of log(*ω*) for subfamily B, subfamily A (without variant 3) and subfamily A/variant 3 sequences, respectively. A fireplot visualizes the posterior log(*ω*) along the sequence using a colour gradient, where a higher posterior density is represented by more intense colour (closer to white) and lower posterior density is represented by less intense colour (closer to red). (d), (e) and (f) depict the posterior probability of positive selection (*y* axis, values 0 to 1) along the codon sequence (*x* axis) for subfamily B, subfamily A (without variant 3) and subfamily A/variant 3 sequences, respectively. Note: variants 2 and 3 differ from subfamily B/variant 1 in length by +4 and +7 bp, respectively, e.g. residue 147 of variant 1 is at position 151 of subfamily A/variant 2 (e), and at position 154 of subfamily A/variant 3 (d).

**Fig. 5.**
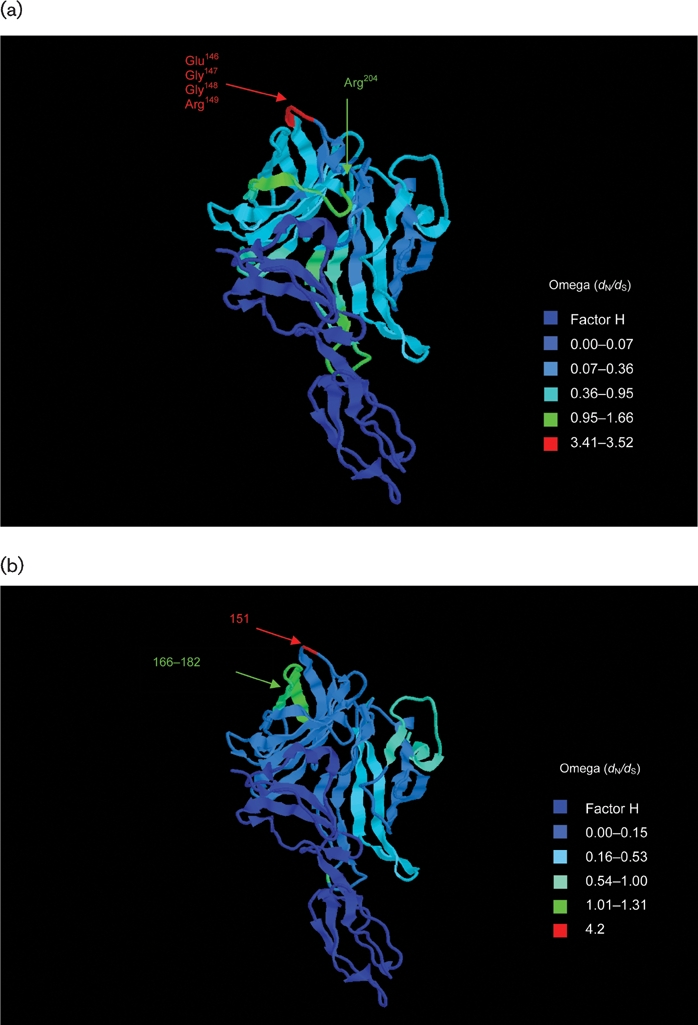

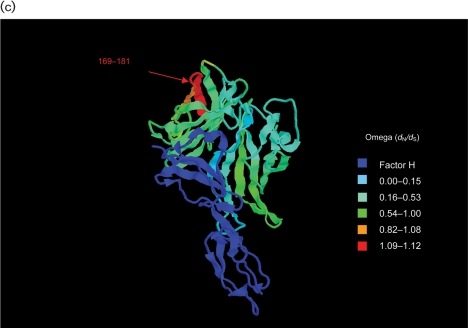
Structure of the fH–fHbp complex (Schneider *et al.*, 2009), with temperature colouring using per-site point estimates of *ω* for (a) subfamily B/variant 1 sequences, (b) subfamily A/variant 2 sequences and (c) subfamily A/variant 3 sequences. Peptides indicated in (a) are putative bactericidal epitopes identified elsewhere (Giuliani *et al.*, 2005; Welsch *et al.*, 2004; Scarselli *et al.*, 2009). In (b) and (c), positively selected sites are indicated. Note: subfamily A/variant 2 and subfamily A/variant 3 differ in length from subfamily B/variant 1 by +4 bp (e.g. Glu^151^ is equivalent to Glu^147^ in variant 1) and +7 bp (e.g. Glu^154^ is equivalent to Glu^147^ in variant 1), respectively.

The fHbp locus had an average *d*_N_* *: *d*_S_ ratio of 0.35, indicating a level of purifying selection against amino acid change. Previous estimates have been 0.51±0.7 (Bambini *et al.*, 2009) and comparable to that of other antigenic genes such as *fetA* (0.314) (Thompson, 2001). Codon-by-codon analysis of selection on the gene was possible using omegamap. Separate analyses for each of the subfamilies/variants, including variant 3, indicated that in the C-terminal region (after ∼318 nt encoding 106 aa) there was diversifying immune selection (*ω* >1) acting on particular areas in each of the subfamilies/variants (Fig. 4a–f). Subfamily B/variant 1 and subfamily A/variant 2 (not including variant 3) shared one positively selected codon (147 and 151 in subfamily B/variant 1 and subfamily A/variant 2, respectively). Subfamily B/variant 1 displayed positive selection at the four codons 146–149 (*ω* 3.41–3.52) and also at codons 195–204 (*ω* 1.02–1.66). Subfamily A/variants 2 and 3 shared the positively selected sites from codons 169–181. The per-site point estimate of *ω* inferred for each of the subfamily/variant isolates was used to colour a 3D pdb file of the solution structure of a complex between a subfamily B/variant 1 GNA1870/fHbp protein and a region of the fH protein (code 2W81) (Schneider *et al.*, 2009). The temperature colouring of the protein enabled the demonstration of the regions under positive selection on the 3D model (Fig. 5a, b and c). These regions did not overlap with residues involved in interactions with the fH molecule (Schneider *et al.*, 2009).

## DISCUSSION

An ideal vaccine candidate provides cross-protection against all variants of a targeted pathogen. To date, proteins suggested as components of meningococcal vaccines either do not elicit protective immune responses or, like fHbp, are variable (Jodar *et al.*, 2002). Consequently, it is important to catalogue this diversity before a vaccine formulation is finalized to ensure maximum vaccine coverage. For fHbp a number of studies have been performed to achieve this (Bambini *et al.*, 2009; Murphy *et al.*, 2009); however, a universal agreed nomenclature is essential to enable comparisons among different studies. Two different fHbp classification schemes have been proposed: one classifies the protein variants of fHbp (referred to as GNA1870) into three variant families, named variants 1, 2 and 3 (Masignani *et al.*, 2003), while the other groups variants of the same protein (referred to as LP2086) into subfamilies A and B (Fletcher *et al.*, 2004). Here, a unified nomenclature is proposed in which unique fHbp peptide and nucleotide sequences are assigned numbers arbitrarily and entered into a database that can be queried and into which new sequences can be deposited (http://neisseria.org/nm/typing/fhbp). Using this nomenclature as a basis, higher-order classifications can be applied without confusion.

Understanding diversity also requires appropriate isolate collections, with a sample frame appropriate to the question addressed. For this reason, the present study investigated the 107 meningococci used to establish multilocus sequence typing (MLST), which includes the globally important disease-associated hyperinvasive meningococcal lineages of all serogroups from the latter half of the twentieth century, which have been extensively characterized (Callaghan *et al.*, 2006; Maiden *et al.*, 1998; Thompson *et al.*, 2003; Urwin *et al.*, 2004). The number of fHbp alleles in this set, 28 encoding 27 peptides, was broadly similar to the number for other surface proteins investigated: PorA (33 alleles encoding 33 peptides); PorB (31 alleles encoding 28 peptides); FetA (33 alleles encoding 31 peptides); and Opa (90 alleles encoding 83 peptides) (Callaghan *et al.*, 2006; Urwin *et al.*, 2004). The diversity of fHbp resolved into two major clusters by the phylogenetic approaches used (Fig. 2), as described previously (Fletcher *et al.*, 2004; Murphy *et al.*, 2009), with evidence of a third group (variant 3) (Masignani *et al.*, 2003). While other variants may be discovered by further studies, especially of carried rather than disease-associated meningococci, comparison with published sequences from various sources (GenBank, http://neisseria.org/nm/typing/fhbp) demonstrated that the 107 isolates included all the major variant clusters of the protein described to date. Of the previously described groups, subfamily B/variant 1 was the most prevalent (60 %) among the 107 isolates. In other studies it accounted for 54–70 % of isolates (Beernink *et al.*, 2007; Fletcher *et al.*, 2004; Jacobsson *et al.*, 2006; Masignani *et al.*, 2003; Murphy *et al.*, 2009; Welsch *et al.*, 2004).

There was evidence that fHbp alleles and consequently variant peptides are generated by horizontal genetic exchange, as is the case for other meningococcal antigens (Bennett *et al.*, 2009; Derrick *et al.*, 1999; Harrison *et al.*, 2008). Furthermore, the gene is also present in *N. gonorrhoeae*, with the gonococcal fHbp sequences described to date belonging to subfamily A/variant 2 group (Fletcher *et al.*, 2004; Masignani *et al.*, 2003). It is possible that subfamily A/variant 3 arose through a recombination event with a DNA fragment donated from another member of the genus *Neisseria*, an idea supported by a neighbour-joining phylogenetic analysis of peptide sequences that clustered the subfamily A/variant 3 sequences between two gonococcal fHbp sequences and other subfamily A/variant 2 sequences (data not shown). Common gene pools have been documented for other *Neisseria* antigens such as PorB2, FetA and TbpB (Bennett *et al.*, 2009; Derrick *et al.*, 1999; Harrison *et al.*, 2008). In the case of PorB and TbpB, different variant classes are thought to have arisen due to inter-species recombination.

While *N. gonorrhoeae* appears to have an fHbp gene, it is known to bind fH via porin proteins (Ngampasutadol *et al.*, 2008). Both meningococci and gonococci have specificity for human fH, which may partly explain their pathogenic restriction in humans (Granoff *et al.*, 2009; Welsch & Ram, 2008). The gene encoding fHbp has also been detected in the commensal species *Neisseria cinerea* and *Neisseria lactamica*, and a potential fHbp peptide has been detected by Western blot analysis (Fletcher *et al.*, 2004; Masignani *et al.*, 2003); however, the distribution of the fHbp gene among the *Neisseria* and its function in the non-pathogenic organisms are yet to be fully elucidated.

Despite the genetic and antigenic diversity of carried populations of meningococci (Caugant & Maiden, 2009; Yazdankhah & Caugant, 2004), most invasive meningococcal disease is caused by a small number of clonal complexes, known as the hyperinvasive lineages (Brehony *et al.*, 2007; Caugant, 2001; Yazdankhah *et al.*, 2004). In common with other variable antigens (Callaghan *et al.*, 2008; Harrison *et al.*, 2008; Trotter *et al.*, 2007; Urwin *et al.*, 2004), the distribution of fHbp variants was not random among clonal complexes, with certain variants more likely to be found in given hyperinvasive lineages, as seen in other isolate collections (Bambini *et al.*, 2009; Jacobsson *et al.*, 2006; Masignani *et al.*, 2003). The ST-32 complex and serogroup A were significantly associated with particular subfamily B/variant 1 peptide alleles (1 and 5 respectively). The ST-11 complex, which can be distinguished from other hyperinvasive lineages by harbouring only *tbpB* isotype I and lacking the *opcA* gene (Claus *et al.*, 2001), was significantly associated with subfamily A and peptide allele type 22. Similarly, stable associations have been observed in serogroup A, X and W-135 meningococci in Africa over a 45-year period (Beernink *et al.*, 2009b). It should be noted, however, that while these associations exist, they are not absolute. For example, while most of the ST-32 complex isolates were peptide allele 1, there was an isolate with the peptide allele 13, and several of the complexes, while they may have a dominant allele type, can also contain other alleles. The ST-41/44 complex in particular was heterogeneous, with multiple allele types (Bambini *et al.*, 2009; Beernink *et al.*, 2007; Jacobsson *et al.*, 2006; Masignani *et al.*, 2003). The reasons for these associations are not fully understood, but models of strain structure in recombining pathogens show that immune selection can, counter-intuitively, lead to the stable associations of antigenic variants characteristic of meningococcal hyperinvasive lineages (Gupta *et al.*, 1996; Callaghan *et al.*, 2008; Buckee *et al.*, 2008).

The availability of protein structures for fHbp, including one with the protein bound to fH (Schneider *et al.*, 2009), allowed analysis of the sequence variability of regions encoding different structural and functional domains. Variation in peptide sequence is present throughout fHbp, rather than being limited to particular variable regions as is the case in the PorA and PorB2 (but not PorB3) porins and FetA. Most fHbp diversity was found in the C-terminal region (∼158 aa in length) of fHbp, while there was less in the N-terminal region (∼105 aa in length), which contained a domain that anchors the protein to the cell membrane (Mascioni *et al.*, 2009). Other invariant regions of the protein are those involved with fH interaction [particularly within subfamilies/variants, although some of the interaction residues show some polymorphism (Schneider *et al.*, 2009)], and residues that make up hydrophobic cores of the *β*-barrels and the points of contact between the N- and C-terminal domains (Mascioni *et al.*, 2009).

The selection pressures acting on fHbp were deduced by means of a Bayesian algorithm, which has a number of advantages over the maximum-likelihood approaches used previously to analyse selection pressures on the PorB protein (Urwin *et al.*, 2002), and the results compared with those obtained in functional studies. Epitope mapping has identified the residue Arg^204^ as being essential for the binding of a bactericidal mAb (Giuliani *et al.*, 2005; Scarselli *et al.*, 2009). Residues also identified as potentially involved in a conformational epitope with Arg^204^ are Glu^146^–Arg^149^ (Cantini *et al.*, 2006; Scarselli *et al.*, 2009; Welsch *et al.*, 2004). Due to their placement and clustering in structural models, it is thought that these residues could make up a bactericidal epitope in the C-terminal region with the potential cooperation of other residues (Cantini *et al.*, 2006; Scarselli *et al.*, 2009), and they have been shown to be placed away from the fH recognition site and therefore may not interfere with fH binding (Schneider *et al.*, 2009). The selection analyses identified these residues as displaying evidence of immune selection on the subfamily B/variant 1 protein, underlining their potential relevance as protective epitopes. One of these residues, 151 (147 in subfamily B/variant 1), also showed evidence of positive selection in subfamily A/variant 2.

Other protective epitopes identified to date include residues 121–122 present in subfamily B/variant 1 proteins, residues between 25 and 59 present in subfamily A/variant 2 and subfamily B/variant 1, and residues between positions 174 and 216 of variant 2 and 3 proteins (Beernink & Granoff, 2008; Beernink *et al.*, 2009a, 2008). Particularly in meningococci expressing fHbp at low levels, these epitopes can induce bactericidal activity by eliciting cooperative pairs of mAbs that also inhibit fH binding, thus increasing complement-mediated activity (Beernink *et al.*, 2008). The selection analysis provided evidence for positive selection in a region that partially overlapped with one of these putative bactericidal epitopes found in the C-terminal region of the subfamily A/variant 2 and subfamily A/variant 3 proteins, i.e. residue 174. These results are very encouraging in that such analyses can be used to predict regions involved in the immune interactions of other bacterial proteins.

Molecular epidemiology has played a major role in the development, implementation and study of meningococcal vaccines (Bjune *et al.*, 1991; Maiden *et al.*, 2008; O'Hallahan *et al.*, 2005; Rodriguez *et al.*, 1999). For candidate protein components it is essential to determine the number of variants required and to identify those likely to provide the broadest possible protection, ideally before a vaccine formulation is tested in humans. Although the use of functional assays is important, nucleotide and peptide sequence diversity give important guides to this process. In the case of fHbp, the existence of multiple variants and the evidence for particular epitopes under immune selection indicate that, as for other meningococcal antigens, it will be important to use vaccine formulations with multiple components to achieve broad coverage, particularly as it has been shown that cross-protection between the two major subfamilies and within subfamily A, variants 2 and 3, is limited (Beernink *et al.*, 2007; Fletcher *et al.*, 2004; Masignani *et al.*, 2003). An alternative strategy is to create chimeric proteins containing domains from the different subfamilies/variants (Beernink & Granoff, 2008). However multivalency is achieved, the optimum number of variants to be used will depend on a combination of molecular epidemiological and functional studies. Furthermore, the lifespan of such vaccines will depend on the dynamics of fHbp evolution in natural populations of meningococci and any possible effects of vaccination on this process. The nomenclature scheme and analytical framework described here should contribute to assembling the information required to answer these questions.

## Figures and Tables

**Table 1.** Study isolates and association with clonal complex, sequence type (ST), year, country, disease, serogroup, peptide allele, nucleotide allele and subfamily/variant

**Isolate name**	**Complex**	**ST**	**Subfamily/variant**	**Year**	**Country**	**Disease**	**Serogroup**	**Peptide allele**	**Nucleotide allele**
393	ST-1 complex	1	SubB/v1	1968	Greece	Carrier	A	4	4
20	ST-1 complex	1	SubB/v1	1963	Niger	Invasive (unspecified/other)	A	4	4
129E	ST-1 complex	1	SubB/v1	1964	West Germany	Invasive (unspecified/other)	A	4	4
254	ST-1 complex	1	SubB/v1	1966	Djibouti	Invasive (unspecified/other)	A	4	4
106	ST-1 complex	1	SubB/v1	1967	Morocco	Invasive (unspecified/other)	A	4	4
6748	ST-1 complex	1	SubB/v1	1971	Canada	Invasive (unspecified/other)	A	4	4
S5611	ST-1 complex	1	SubB/v1	1977	Australia	Invasive (unspecified/other)	A	4	4
79126	ST-1 complex	3	SubB/v1	1979	China	Invasive (unspecified/other)	A	38	37
79128	ST-1 complex	3	SubB/v1	1979	China	Invasive (unspecified/other)	A	38	37
371	ST-1 complex	1	SubB/v1	1980	India	Invasive (unspecified/other)	A	4	4
322/85	ST-1 complex	2	SubB/v1	1985	East Germany	Invasive (unspecified/other)	A	14	14
120M	ST-1 complex	1	SubB/v1	1967	Pakistan	Meningitis and septicaemia	A	4	4
139M	ST-1 complex	1	SubB/v1	1968	Philippines	Unspecified	A	1	1
BZ 133	ST-1 complex	1	SubB/v1	1977	Netherlands	Invasive (unspecified/other)	B	4	4
890326	ST-103 complex	28	SubA/v2	1989	Netherlands	Invasive (unspecified/other)	Z	25	25
NG P20	ST-11 complex	11	SubB/v1	1969	Norway	Invasive (unspecified/other)	B	5	5
D1	ST-11 complex	11	SubA/v2	1989	Mali	Carrier	C	22	22
M597	ST-11 complex	11	SubA/v2	1988	Israel	Invasive (unspecified/other)	C	22	22
BRAZ10	ST-11 complex	11	SubA/v2	1976	Brazil	Unspecified	C	22	22
F1576	ST-11 complex	11	SubA/v2	1984	Ghana	Unspecified	C	22	22
500	ST-11 complex	11	SubA/v2	1984	Italy	Unspecified	C	22	22
MA-5756	ST-11 complex	11	SubA/v3	1985	Spain	Unspecified	C	67	54
90/18311	ST-11 complex	11	SubA/v2	1990	Scotland	Unspecified	C	22	22
L93/4286	ST-11 complex	11	SubA/v2	1993	England	Invasive (unspecified/other)	C	95	92
38VI	ST-11 complex	11	SubA/v3	1964	USA	Unspecified	B	98	95
860800	ST-167 complex	29	SubA/v2	1986	Netherlands	Invasive (unspecified/other)	Y	23	23
EG 328	ST-18 complex	18	SubB/v1	1985	East Germany	Invasive (unspecified/other)	B	37	36
EG 327	ST-18 complex	19	SubA/v2	1985	East Germany	Invasive (unspecified/other)	B	33	41
1000	ST-18 complex	20	SubB/v1	1988	USSR	Invasive (unspecified/other)	B	5	5
528	ST-18 complex	18	SubB/v1	1989	USSR	Invasive (unspecified/other)	B	37	36
E26	ST-198 complex	39	SubA/v3	1988	Norway	Carrier	X	94	91
A22	ST-22 complex	22	SubA/v2	1986	Norway	Carrier	W-135	16	16
71/94	ST-23 complex	23	SubA/v2	1994	Norway	Invasive (unspecified/other)	Y	25	25
297-0	ST-254 complex	49	SubB/v1	1987	Chile	Carrier	B	13	13
NG F26	ST-269 complex	14	SubA/v2	1988	Norway	Carrier	B	19	19
NG 6/88	ST-269 complex	13	SubA/v2	1988	Norway	Invasive (unspecified/other)	B	32	40
44/76	ST-32 complex	32	SubB/v1	1976	Norway	Invasive (unspecified/other)	B	1	1
NG 080	ST-32 complex	32	SubB/v1	1981	Norway	Invasive (unspecified/other)	B	1	1
NG144/82	ST-32 complex	32	SubB/v1	1982	Norway	Invasive (unspecified/other)	B	1	1
BZ 83	ST-32 complex	34	SubB/v1	1984	Netherlands	Invasive (unspecified/other)	B	1	1
EG 329	ST-32 complex	32	SubB/v1	1985	East Germany	Invasive (unspecified/other)	B	1	1
BZ 169	ST-32 complex	32	SubB/v1	1985	Netherlands	Invasive (unspecified/other)	B	1	1
NG PB24	ST-32 complex	32	SubB/v1	1985	Norway	Invasive (unspecified/other)	B	1	1
8680	ST-32 complex	32	SubB/v1	1987	Chile	Invasive (unspecified/other)	B	13	13
204/92	ST-32 complex	33	SubB/v1	1992	Cuba	Invasive (unspecified/other)	B	1	1
196/87	ST-32 complex	32	SubB/v1	1987	Norway	Unspecified	C	1	1
E32	ST-334 complex	31	SubA/v2	1988	Norway	Carrier	Z	19	19
SWZ107	ST-35 complex	35	SubA/v2	1986	Switzerland	Invasive (unspecified/other)	B	16	16
NG E31	ST-364 complex	15	SubA/v2	1988	Norway	Carrier	B	34	43
DK 353	ST-37 complex	37	SubA/v2	1962	Denmark	Invasive (unspecified/other)	B	24	24
BZ 232	ST-37 complex	38	SubA/v2	1964	Netherlands	Invasive (unspecified/other)	B	24	24
A4/M1027	ST-4 complex	4	SubB/v1	1937	USA	Invasive (unspecified/other)	A	5	5
10	ST-4 complex	4	SubB/v1	1963	Burkina Faso	Invasive (unspecified/other)	A	5	5
26	ST-4 complex	4	SubB/v1	1963	Niger	Invasive (unspecified/other)	A	5	5
255	ST-4 complex	4	SubB/v1	1966	Burkina Faso	Invasive (unspecified/other)	A	5	5
S3131	ST-4 complex	4	SubB/v1	1973	Ghana	Invasive (unspecified/other)	A	5	5
690	ST-4 complex	4	SubB/v1	1980	India	Invasive (unspecified/other)	A	5	5
C751	ST-4 complex	4	SubB/v1	1983	Gambia	Invasive (unspecified/other)	A	5	5
1014	ST-4 complex	4	SubB/v1	1985	Sudan	Invasive (unspecified/other)	A	5	5
2059001	ST-4 complex	4	SubB/v1	1990	Mali	Invasive (unspecified/other)	A	5	5
D8	ST-4 complex	4	SubB/v1	1990	Mali	Invasive (unspecified/other)	A	5	5
243	ST-4 complex	4	SubB/v1	1966	Cameroon	Unspecified	A	5	5
NG H36	ST-41/44 complex	47	SubA/v2	1988	Norway	Carrier	B	19	19
NG E30	ST-41/44 complex	44	SubA/v2	1988	Norway	Carrier	B	32	42
BZ 147	ST-41/44 complex	48	SubB/v1	1963	Netherlands	Invasive (unspecified/other)	B	4	4
BZ198	ST-41/44 complex	41	SubB/v1	1986	Netherlands	Invasive (unspecified/other)	B	5	5
88/03415	ST-41/44 complex	46	SubB/v1	1988	Scotland	Invasive (unspecified/other)	B	14	14
91/40	ST-41/44 complex	42	SubB/v1	1991	New Zealand	Invasive (unspecified/other)	B	14	14
400	ST-41/44 complex	40	SubB/v1	1991	Austria	Invasive (unspecified/other)	B	14	14
AK50	ST-41/44 complex	41	SubB/v1	1992	Greece	Invasive (unspecified/other)	B	4	4
M-101/93	ST-41/44 complex	41	SubB/v1	1993	Iceland	Invasive (unspecified/other)	B	4	4
931905	ST-41/44 complex	41	SubB/v1	1993	Netherlands	Invasive (unspecified/other)	B	14	14
50/94	ST-41/44 complex	45	SubB/v1	1994	Norway	Invasive (unspecified/other)	B	4	4
M40/94	ST-41/44 complex	41	SubB/v1	1994	Chile	Invasive (unspecified/other)	B	35	32
N45/96	ST-41/44 complex	41	SubB/v1	1996	Norway	Invasive (unspecified/other)	B	1	1
NG H15	ST-41/44 complex	43	SubA/v2	1988	Norway	Carrier	B	19	19
80049	ST-5 complex	5	SubB/v1	1963	China	Carrier	A	39	38
F4698	ST-5 complex	5	SubB/v1	1987	Saudi Arabia	Carrier	A	14	14
153	ST-5 complex	5	SubA/v2	1966	China	Invasive (unspecified/other)	A	22	22
154	ST-5 complex	6	SubB/v1	1966	China	Invasive (unspecified/other)	A	36	35
S4355	ST-5 complex	5	SubB/v1	1974	Denmark	Invasive (unspecified/other)	A	5	5
7891	ST-5 complex	5	SubB/v1	1975	Finland	Invasive (unspecified/other)	A	5	5
11-004	ST-5 complex	5	SubB/v1	1984	China	Invasive (unspecified/other)	A	5	5
H1964	ST-5 complex	5	SubB/v1	1987	UK	Invasive (unspecified/other)	A	5	5
92001	ST-5 complex	7	SubB/v1	1992	China	Invasive (unspecified/other)	A	5	5
14/1455	ST-5 complex	5	SubB/v1	1970	USSR	Unspecified	A	14	14
IAL2229	ST-5 complex	5	SubB/v1	1976	Brazil	Unspecified	A	5	5
F6124	ST-5 complex	5	SubA/v2	1988	Chad	Invasive (unspecified/other)	A	25	25
860060	ST-750 complex	24	SubA/v3	1986	Netherlands	Invasive (unspecified/other)	X	84	69
BZ 10	ST-8 complex	8	SubA/v2	1967	Netherlands	Invasive (unspecified/other)	B	18	39
B6116/77	ST-8 complex	10	SubA/v2	1977	Iceland	Invasive (unspecified/other)	B	16	16
BZ 163	ST-8 complex	9	SubA/v2	1979	Netherlands	Invasive (unspecified/other)	B	16	16
G2136	ST-8 complex	8	SubA/v2	1986	England	Invasive (unspecified/other)	B	16	16
AK22	ST-8 complex	8	SubA/v2	1992	Greece	Invasive (unspecified/other)	B	16	16
SB25	ST-8 complex	8	SubA/v2	1990	South Africa	Invasive (unspecified/other)	C	16	16
94/155	ST-8 complex	66	SubA/v2	1994	New Zealand	Invasive (unspecified/other)	C	16	16
312 901	ST-8 complex	8	SubA/v2	1996	England	Invasive (unspecified/other)	C	16	16
CN100	Unassigned	21	SubB/v1	1941	England	Invasive (unspecified/other)	A	4	4
NG E28	Unassigned	26	SubB/v1	1988	Norway	Carrier	B	14	14
3906	Unassigned	17	SubA/v2	1977	China	Invasive (unspecified/other)	B	18	39
NG 4/88	Unassigned	30	SubB/v1	1988	Norway	Invasive (unspecified/other)	B	4	4
NG 3/88	Unassigned	12	SubA/v3	1988	Norway	Invasive (unspecified/other)	B	96	93
NG G40	Unassigned	25	SubA/v2	1988	Norway	Carrier	B	24	24
EG 011	Unassigned	36	SubA/v2	1986	East Germany	Invasive (unspecified/other)	B	24	24
NG H41	Unassigned	27	SubA/v2	1988	Norway	Carrier	B	25	25
DK 24	Unassigned	16	SubA/v2	1940	Denmark	Invasive (unspecified/other)	B	97	94
NG H38	Unassigned	36	SubA/v2	1988	Norway	Carrier	B	24	24

## Abbreviations

- fH, factor H
- fHbp, factor H-binding protein
- *ω*, omega (*d*_N_ *d*_S_)
- ST, sequence type
